# Positive selection of *AS3MT* to arsenic water in Andean populations

**DOI:** 10.1016/j.mrfmmm.2015.07.007

**Published:** 2015-07-29

**Authors:** Christina A. Eichstaedt, Tiago Antao, Alexia Cardona, Luca Pagani, Toomas Kivisild, Maru Mormina

**Affiliations:** a Division of Biological Anthropology, University of Cambridge, Cambridge CB2 1QH, Cambridgeshire, UK; b Department of Vector Biology, Liverpool School of Tropical Medicine, Liverpool L3 5QA, Lancashire, UK; c Wellcome Trust Sanger Institute, Hinxton CB10 ISA, Cambridgeshire, UK; d Center for Pulmonary Hypertension, Thoraxclinic at the University Hospital Heidelberg, 69126 Heidelberg, Baden-Württemberg, Germany; e Faculty of Humanities and Social Sciences, University of Winchester, Winchester SO22 4NR, Hampshire, UK

**Keywords:** Arsenic drinking water, Collas, Puna, Methyltransferase, Calchaquíes

## Abstract

Arsenic is a carcinogen associated with skin lesions and cardiovascular diseases. The Colla population from the Puna region in Northwest Argentinean is exposed to levels of arsenic in drinking water exceeding the recommended maximum by a factor of 20. Yet, they thrive in this challenging environment since thousands of years and therefore we hypothesize strong selection signatures in genes involved in arsenic metabolism. We analyzed genome-wide genotype data for 730,000 loci in 25 Collas, considering 24 individuals of the neighbouring Calchaquíes and 24 Wichí from the Gran Chaco region in the Argentine province of Salta as control groups. We identified a strong signal of positive selection in the main arsenic methyltransferase *AS3MT* gene, which has been previously associated with lower concentrations of the most toxic product of arsenic metabolism monomethylarsonic acid. This study confirms recent studies reporting selection signals in the *AS3MT* gene albeit using different samples, tests and control populations.

## 1 Introduction

High levels of arsenic in drinking water are found in countries all over the world [1]. Arsenic mainly originates from minerals in the ground and enters the food chain through drinking water and food sources such as crop plants [2]. Anthropogenic actions like mining and pesticide use contribute to elevated levels of arsenic [3].

Long-term exposure to arsenic can result in cancer, skin lesions, as well as cardiovascular and pulmonary diseases [4]. However, not only at a later stage in life but already at an early age arsenic exposure can have drastic consequences. Arsenic can cross the placental barrier and thus affect the foetal development. Arsenic alters immune response modulator concentrations measured in breast milk [5] as well as in newborn cord blood [6]. Subsequently, high arsenic intake by drinking water in early childhood increases the risk of respiratory infections and diarrhea in infants [7] as well as liver cancer associated mortality [8]. This suggests that populations exposed to high levels of arsenic over long periods of time may possess some kind of protection against arsenic toxicity.

In the body, inorganic arsenic is modified to monomethylarsonic acid (MMA) and subsequently to dimethylarsinic acid (DMA) [9] by methyltransferases. The second reaction occurs much faster due to an increased substrate affinity of the enzyme for MMA and therefore DMA is the predominant end product of arsenic metabolism [10]. Inorganic arsenic, MMA and DMA are excreted in the urine and can be used to measure arsenic metabolism. The most toxic arsenic product is MMA; thus, the first step in the arsenic metabolism is considered to be rather an activation than a detoxification of arsenic [11]. Hence, low levels of MMA in comparison to DMA in urine are beneficial to reduce its toxicity [12].

In the highlands of Northwest Argentina, the Puna, high levels of arsenic in water have been present since many thousands of years [13]. In some locations levels exceed the maximum safe level set by the WHO of 10 µg/l by a factor 20 [9]. San Antonio de los Cobres, in the heart of the Puna region, is one of such localities [9,14]. Yet, its inhabitants show unusually low levels of excreted MMA metabolite relative to DMA and inorganic arsenic [9]. In agreement with this observation, Puna highlanders show increased frequencies of arsenic methyltransferase (*AS3MT*) alleles that have been associated with low MMA urine concentrations [15–17]. Allele differences in Collas were associated with enzyme expression levels [16] and resulting concentrations of arsenic metabolites. Lower levels of MMA were found in Collas compared to Bangladeshi [15], Chinese or Tibetans [18] exposed to permanently elevated arsenic levels in drinking water. Genes responsible for the metabolism of arsenic, therefore, may have been targets of strong positive selection among these populations. Levels of MMA and DMA have been recently associated with various SNPs near *AS3MT* in women from the Colla population of San Antonio de los Cobres in the Argentinean Puna region [19]. Moreover, an allele frequency based selection test applied on genome-wide genotype data in the same study suggested *AS3MT* as one of the main candidates of selection in this population.

In this study, we investigate the strength of the selection pressure exerted by elevated arsenic levels on the genome of a different subset of men and women from the Colla population from San Antonio de los Cobres and surrounding villages. We use two neighboring groups, the Calchaquí and the Wichí as control populations. We also assessed genome-wide genotype data using distinct allele frequency based selection test and were able to confirm strong signatures within and near the *AS3MT* gene, thus underlining the key role of this gene in the adaptation to environmental arsenic.

## 2 Materials and methods

### 2.1 Subjects and ethical approval

**Fig. 1:**
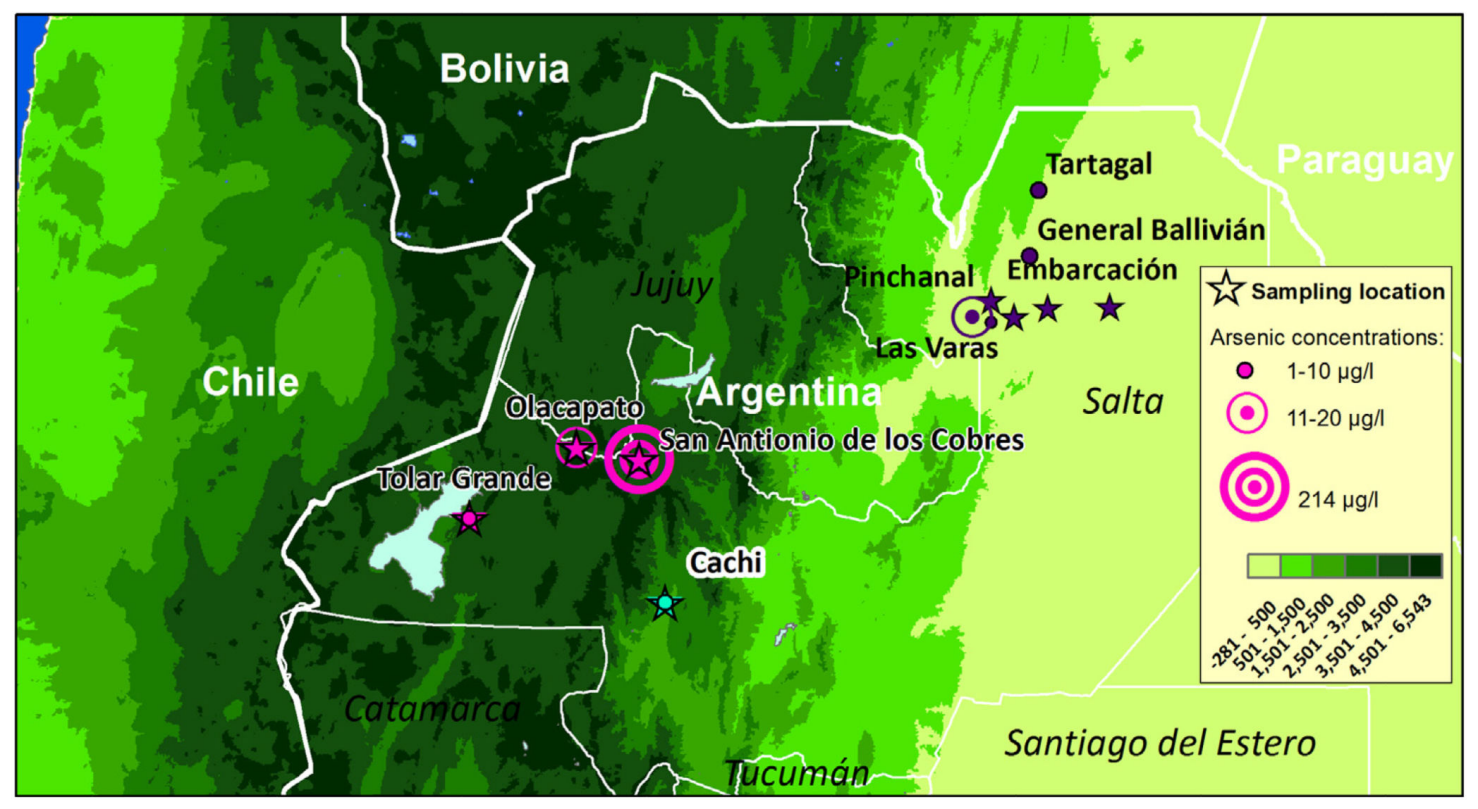
Sampling locations and arsenic levels in the province of Salta, Argentina. Stars denote sampling locations, circles levels of arsenic. Sampling locations of the Wichí population in the Gran Chaco region are (left to right): Embarcación, Carboncito, Misión Chacheña, Dragones (purple stars). Arsenic concentrations in surrounding locations were measured to be: Las Varas 0 µg/l, Pinchanal 19.5 µg/l, General Ballivián 4 µg/l, Tartagal 2.3 µg/l [22]. Calchaquíes originated from Cachi (turquoise star) with an arsenic level of 3.1 µg/l [22]. Collas (pink stars) were sampled in: San Antonio de los Cobres (arsenic level: 214 µg/l), Tolar Grande (arsenic level: 4 µg/l) and Olacapato (arsenic level of 12 µg/l) [14].

Individuals with indigenous ancestry from three regions of the Northwestern Argentinean province of Salta were recruited to participate in this study in April 2011: (1) Collas from the Andean Plateau or Puna (>3500 m), (2) Calchaquíes from Cachi in the Calchaquí valleys at 2300 m and (3) Wichí from the plains of the Gran Chaco region near Embarcación (Fig. 1). We used our previously published data [20,21] for 730,525 single nucleotide polymorphisms (SNPs) genotyped in 25 Collas (11 men, 14 women), 24 Calchaquíes (10 men, 14 women) and 24 Wichí (12 men, 12 women). In the Colla sample, 16 individuals were from San Antonio de los Cobres, where arsenic levels reach 214 µg/l [14]; 7 were from Tolar Grande with arsenic levels of 4 µg/l and one individual was from Olacapato, where arsenic levels are 12 µg/l. Arsenic concentrations for the exact sampling locations in the Gran Chaco regions were not available, however in surrounding locations arsenic concentrations were measured to be: Las Varas 0 µg/l, Pinchanal 19.5 µ/l, General Ballivián 4 µg/l, Tartagal 2.3 µg/l [22]. Concentrations in Cachi (Río Las Trancas) were 3.1 µg/l [22].

Only healthy unrelated adults who gave written informed consent were included in the study. The study was approved by the University of East Anglia Research Ethics Committee, the Ministry of Health of the Province of Salta (Ministerio de Salud Pública, Salta, Argentina) and the University of Cambridge’s Human Biology Research Ethics Committee (HBREC.2011.01).

### 2.2 Genotype data analysis

In total, 726,090 SNPs passed a genotype call rate of >98% and were included in downstream analyses [20,21]. Two tests for positive selection were employed to analyze genome-wide signatures of arsenic adaptation. The pairwise fixation index (*F*_ST_) was used as a measure of population differentiation [23] between Collas and Wichí, and between Calchaquí and Wichí using the programme GENEPOP [24]. We defined genomic windows of 200 kb and used maximal *F*_ST_ values to rank them. Only the top 1% was considered for analyses. Because the direction of the pairwise *F*_ST_ signatures cannot be determined (i.e. the signal can be due to extreme allele frequencies in either of the two populations), we also used the population branch statistic (PBS) to pinpoint allele differentiation to the population of interest [25,26]. PBS is based on pairwise *F*_ST_ of three populations. Collas and Calchaquíes were each compared to Wichí and Eskimos [27]. Eskimos were chosen as the closest non-American outgroup genotyped on the same genotyping platform as Collas, Calchaquíes and Wichí. They originated from Novoe Chaplino, Chukotka Autonomous Okrug in Northeast Siberia [27]. PBS was calculated following Yi et al. [25] using a modified approach from Pickrell et al. [28] for 100 kb windows ranked by maximum PBS values [21]. Regional analysis of linkage disequilibrium was carried out with HaploView 4.2 [29].

As the first step in functional interpretation of the results of selection scanning, we compiled a list of genes known to be involved in arsenic metabolism. We included genes from three different sources: (a) from the Gene Ontology (GO) database AmiGO we extracted genes that matched the search keyword ‘arsen’ to include metabolites of arsenic such as arsenate and arsenite [30]; (b) from the gene information database GeneCards [31] we extracted genes associated with any compound containing the keyword ‘arsen’; (c) additional methyltransferases were extracted from literature [15,32]. The final candidate gene list consisted of 35 unique genes (Table A1). The selection test results were subsequently screened for these 35 candidate genes of arsenic metabolism.

Allele frequency differences between the three populations were assessed with One Way Analysis of Variance (ANOVA) implemented in the Statistical Package for Social Sciences (SPSS), version 20.

## 3 Results

**Fig. 2:**
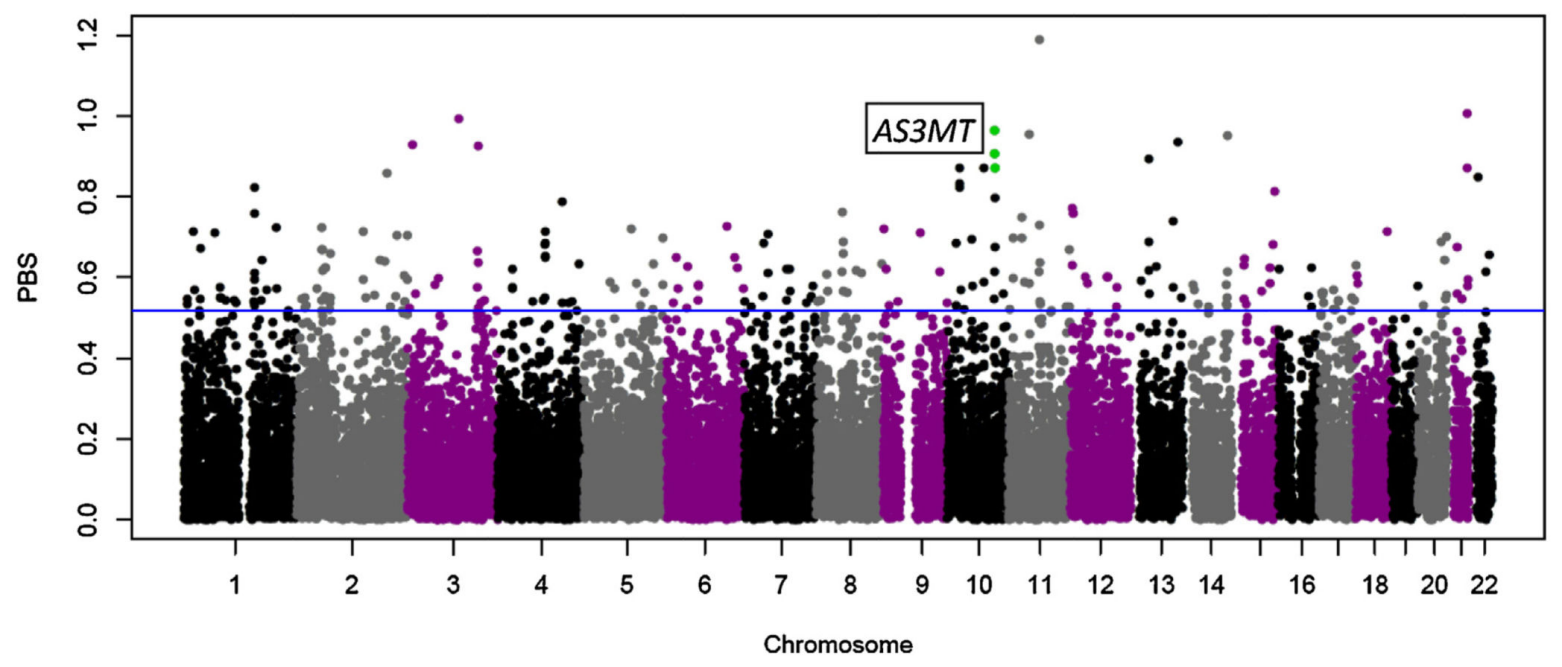
Window PBS scores across the genome in Collas. The blue line indicates the top 1% of hits. The fourth highest cluster overall is found on chromosome 10. The green circles indicate the PBS hits ±1 Mb of *AS3MT.* The highest scoring SNP overall lies on chromosome 11 and falls within a gene free region. The hit on chromosome 21 is located within *CBS*, which regulates cerebral blood flow velocity.

We conducted whole genome scans in Collas and Calchaquíes to identify genetic loci that showed higher than genome-wide average allelic differences between populations (*F*_ST_ and PBS tests). These scans highlighted the arsenic methyltransferase (*AS3MT*) gene as being highly differentiated in the Colla population. The gene was among the top 15 windows in PBS of Colla highlanders (Fig. 2) and among the top 40 windows of pairwise *F*_ST_ between Collas and Wichí.

**Fig. 3:**
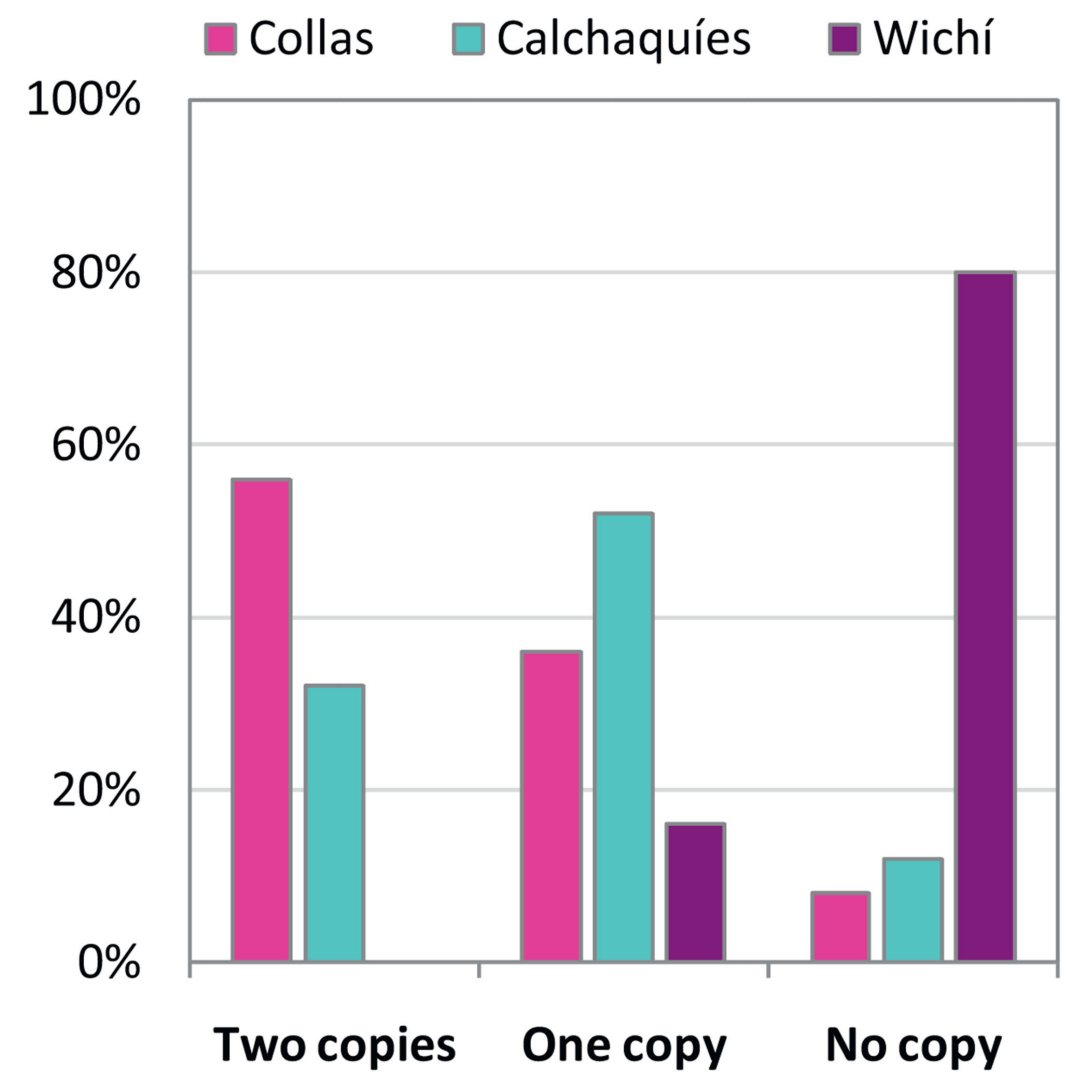
Distribution of beneficial CG alleles in Argentinean populations. The majority of Collas has two copies of the beneficial CG alleles (rs1046778, rs7085104) within and near *AS3MT*, while Calchaquíes mainly carry one copy of the specific alleles. In Wichí most individuals have no copy of the beneficial alleles. Allele frequencies differ significantly between Collas and Wichí and Calchaquíes and Wichí (p <0.001).

The pairwise *F*_ST_ signal was exclusively driven by two SNPs, one within the *AS3MT* gene (rs1046778, *F*_ST_ = 0.606) and another one 1 kb upstream of *AS3MT* (rs7085104, *F*_ST_ = 0.564; genome-wide mean *F*_ST_ = 0.041). Specific variants of these alleles have been associated with beneficial arsenic metabolism [15]. The C allele of the T/C SNP rs1046778 was more frequent in Collas than in Wichí (74% and 8% respectively). The G allele of the G/A SNP rs7085104 was also prevalent in Collas (78% compared to 15% in Wichí). This is consistent with previously reported frequencies for these alleles [15], which have been associated with overall decreased expression of *AS3MT* and lower excreted MMA levels [15]. Engström et al. showed a 175% increase of *AS3MT* expression in homozygous carriers of the T allele at the rs1046778 locus compared to homozygotes of the C allele. Overall, 92% of Collas were at least heterozygous for the C and G allele on the same chromosomal strand corresponding to both functionally advantageous alleles (Fig. 3). The percentage of homozygotes for both beneficial alleles is decreased in individuals from the Calchaquí valley, but not significantly. However, allele frequencies in both Collas and Calchaquíes differed significantly from Wichí (p < 0.001, ANOVA). A recent study using a dataset with greater SNP density, however, could not identify these two previously highlighted SNPs among the top 20 SNPs associated with MMA or DMA concentrations in 124 women from San Antonio de los Cobres [19].

**Fig. A1:**
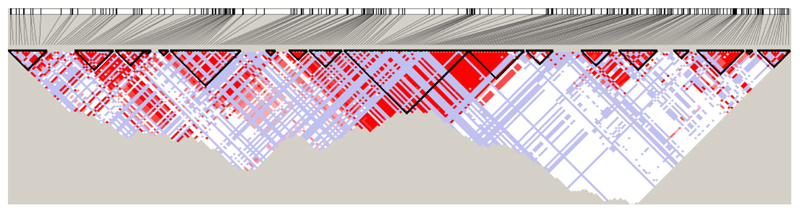
Haplotype analysis of SNPs ±1 Mb around AS3MT. The region includes SNPs located 1 Mb up and downstream of *AS3MT* on chromosome 10 between positions 104, 629, 210–104, 661, 656. The central largest triangle includes *AS3MT* and spans 499 kb. SNPs are represented on the top and the linkage disequilibrium (LD) degree is displayed below by colour. Red squares represent strong LD (*D*’ = 1) with a high logarithm of odds (LOD) score, i.e. the probability of linkage between 2 loci is very high; purple: high *D*’, low LOD score; white: low *D*’ and low LOD score. Black triangles indicate haplotype blocks calculated by the programme Haploview [29]. Only heterozygous SNPs are displayed.

In agreement with our *F*_ST_ results, PBS comparisons of Collas, Wichí, and Eskimos highlighted a window containing *AS3MT* and two neighbouring genes, *CNNM2* and *WBP1L* (Table A2). However, this test identified a different set of SNPs than *F*_ST_ in the surrounding region of *AS3MT.* The SNP (rs12221064) nearest to the gene region identified by PBS was located 15 kb downstream of *AS3MT* within *CNNM2* (Table A2) and ranked 11^th^. Other high-ranking SNPs included rs17115100, within *CYP17A1*, 38 kb upstream of *AS3MT* (ranking 4^th^), and rs11191514 within *CNNM2*, 112 kb downstream (ranking 10^th^) (Table A2). The recent study by Schlebusch and colleagues associated rs17115100 and rs11191514 with percentage of MMA in urine and rs17115100 also with percentage of DMA in urine [19]. The allele frequency *F*_ST_ based selection test used by these authors (LSBL, locus specific branch length test) also highlighted *AS3MT* as top candidate of selection in the Colla population with Peruvians and Colombians as control populations. We previously reported haplotype based selection tests in Collas [21] but *AS3MT* was not among the top 1% haplotypes. However, a regional haplotype analysis 1 Mb up and downstream of *AS3MT* identified a haplotype block of 499 kb containing *AS3MT* (Fig. A1).

We repeated the PBS test using a neighbouring population to the Collas, the Calchaquíes, comparing it to Wichí and Eskimos. This test identified the same upstream SNP (rs17115100), albeit the SNP containing window ranked much lower (50^th^). While the top 1% *F*_ST_ results from Calchaquíes lacked *AS3MT*, it contained another gene from the candidate gene list, the cyclin-dependent kinase inhibitor 1A (*CDKN1A*) gene (rank 60). This kinase inhibitor is a modulator of the cell cycle and was inferred by orthologs to respond to an arsenic-containing substance (G0:0046685, evidence: inferred through electronic annotation).

*AS3MT* was the only of the 35 arsenic candidate genes (Table A1) showing a signature of selection with two selection tests in the same population.

## 4 Discussion

High concentrations of arsenic in drinking water represent a strong environmental stressor, driving significant adaptive change in the highland populations of the Argentinean Puna. In this study, *AS3MT* was identified by our genome-wide scans as the main outcome of positive selection. Alleles within or nearby this gene are highly differentiated and appear within the top 1% of ca. 13,000 windows across the genome. *AS3MT* had not been identified previously the top 1% of two haplotype based tests (integrated haplotype score, iHS and cross population extended haplotype homozygosity, XP-EHH) in Collas [21]. However, the minimum SNP density for iHS in a 200 kb window was not reached in the respective window containing the gene; therefore, no iHS test statistic could be calculated. XP-EHH neither highlighted the respective window as a particular long high frequency haplotype [21].

Thus, the selection signature of *AS3MT* was not identified by our previous haplotype based tests [21] but only by allele frequency based tests. Though a similar study also failed to identify a strong selection signal with iHS, it reported the average iHS values in a 1 Mb window around *AS3MT to* be among the top 3%. In both studies, allele frequency based tests lead to more conclusive results, suggesting selection from standing variation in the ancestral population prior to the exposure to high arsenic concentrations. The alleles identified by our present study have been functionally evaluated and associated with reduced MMA concentrations in the Colla population of San Antonio de los Cobres [15,19]. High concentrations of MMA are associated with arsenic related diseases [12]; thus, the metabolism of Argentine Puna inhabitants seems fine-tuned to reduce toxic MMA [9]. Only *AS3MT* could be highlighted from the arsenic candidate gene list by two selection tests using a genome-wide genotype approach. An alternative arsenic methyltransferase *N6AMT1*, which was also associated with lower MMA in Collas [33], did not reach genome-wide significance (data not shown).

The findings of our study are therefore well in agreement with a previous recent report [19] suggesting selection pressure from arsenic water in the Colla population, albeit analyzing different individuals, using distinct control populations and different *F*_ST_ based selection tests (PBS and *F*_ST_ instead of LSBL). Alleles both within and around *AS3MT* appear to be the target of strong positive selection. The SNPs around *AS3MT* could be in linkage with a regulatory or functional variant or could itself influence *AS3MT* expression. An analysis of the region revealed a haplotype block of approximately 499 kb around the gene region (Fig. A1), thus suggesting selection of surrounding SNPs. Schlebusch et al. [19] also highlighted selection signatures outside the coding region of the *AS3MT* gene. Whole genome scans have the potential to reveal more distantly located loci with functional relevance, which may be overlooked by targeted resequencing of specific gene regions. Besides reporting strong signatures around *AS3MT*, we also highlighted adjacent genes, such as *CMMN2* or *CYP17A1*, and cannot unequivocally exclude that these may also contribute in particular to the PBS selection signal. However, considering the high *F*_ST_ scores within *AS3MT*, the functional relevance of this gene in the arsenic metabolism and association of overrepresented alleles in Collas with its expression [15], *AS3MT* is a likely candidate of selection. Nevertheless functional *in vitro* and *in vivo* studies of alleles are necessary for a more conclusive interpretation. In this regard, it is worth noting that the neighboring genes *CNNM2* and *WPB1L* (Table A2), have been shown to be differentially methylated in the Colla population [16]. Since methylation reduces gene expression, a decreased level of the arsenic methyltransferase in peripheral blood was observed [16]. The reduced expression of this enzyme is associated with lower levels of MMA [15] and thus most likely beneficial in an environment with elevated arsenic concentrations.

It is interesting to note, that the *F*_ST_ values for the two highlighted alleles within 1 kb upstream of the *AS3MT* gene were 10 fold higher (0.606 and 0.564) than the gene’s average *F*_ST_ of 0.053 calculated in another study, which compared Collas to indigenous Peruvians [17]. This underlines the extreme allele differentiation of two functionally associated SNPs compared to the complete gene region.

Significant differences in the allele frequency of *AS3MT* were also observed between Calchaquíes, Wichí and Eskimos, even though arsenic levels in ground water in the Calchaquí region are lower than those in the Puna [22]. The selection signature of *AS3MT* ranks lower in Calchaquíes than in Collas albeit still among the top 1%, thus, implying either a reduced selection pressure in the Calchaquí population or gene flow from Collas [20]. Calchaquíes also show a selection signature around *CDKN1A*, as indicated by pairwise *F*_ST_, although this signature is less strong than that of *AS3MT* in Collas. The functional significance of this cell cycle regulator for arsenic metabolism remains to be clarified.

In summary, our study confirms previous claims that positive selection has shaped allele frequencies of *AS3MT* to allow adaptation to the extremely toxic element arsenic [19]. We show signatures of positive selection driving allele frequencies in Collas and, to a smaller degree, in the neighboring Calchaquí population. Selected alleles have enabled these populations to thrive for thousands of years despite their constant exposure to high levels of arsenic in drinking water.

## 5 Conclusion

The toxicant arsenic was shown to shape allele frequencies of the main arsenic methyltransferase in Argentinean Collas and Calchaquíes. This study confirms recent findings highlighting the strong selection pressure of the environmental carcinogen arsenic at a genome-wide level. This suggests that natural selection has given carriers of beneficial alleles higher reproductive success to thrive despite the daily consumption of high levels of arsenic.

## Appendix A

**Table A1:** Arsenic detoxification associated candidate genes.

Source	Gene	Name
AmiGO: “arsen” associated ontology	*ABCC2*	ATP-binding cassette sub-family C member 2
	*AS3MT*	Methylarsonite methyltransferase (arsenite methyltransferase)
	*ASNA1*	Arsa (Bacterial) arsenite transporter, Atp-binding, homolog 1
	*CDKN1A*	Cyclin-dependent kinase inhibitor 1
	*CPEB2*	Cytoplasmic polyadenylation element-binding protein 2
	*CPOX*	Coproporphyrinogen oxidase
	*CYP1A1*	Cytochrome P1-450, dioxin-inducible
	*DDX3X*	DEAD (Asp-Glu-Ala-Asp) box helicase 3, X-linked
	*FECH*	Errochelatase
	*GCLC*	Glutamate-cysteine ligase, catalytic subunit
	*GLRX2*	Glutaredoxin 2
	*GSTO1*	Glutathione S-transferase omega-1 (monomethylarsonic acid reductase)
	*GSTO2*	Glutathione S-transferase omega-2 (monomethylarsonic acid reductase)
	*HMOX1*	Heme Oxygenase 1
	*MKNK2*	MAP kinase interacting serine/threonine kinase 2
	*PPIF*	Peptidylprolyl isomerase F (cyclophilin F)
	*PTEN*	Phosphatidylinositol-3,4,5-trisphosphate 3-phosphatase
	*RBM4*	RNA-binding motif protein 4
	*RNF4*	RING finger protein 4
	*SLC34A1*	Solute Carrier family 34 member 1
	*SRRT*	Serrate RNA effector molecule homolog (arsenite-resistance protein 2)
	*TNFRSF11B*	Tumor necrosis factor receptor superfamily member 11B
	*UROS*	Uroporphyrinogen-Ill synthase
	*ZFAND1*	Zinc finger, AN1-type domain 1
	*ZFAND2A*	Zinc finger, AN1-type domain 2A (arsenite inducible RNA Associated protein)
	*ZFAND2B*	Zinc finger, AN1-type 2B (arsenite-inducible RNA-associated protein-like protein)
Gene cards: “arsen” associated gene name	*METTL18*	Methyltransferase-like protein 18 (arsenic-transactivated protein 2)
	*POLE3*	Polymerase (DNA directed), epsilon 3 (arsenic-transactivated protein)
	*SERPINH1*	Serine (or cysteine) proteinase inhibitor, clade H (arsenic-transactivated protein 3)
	*SPDL1*	Spindly homolog (drosophila) (arsenite-related gene 1 protein)
Literature: arsenic associated methyl-transferases	*BHMT*	Betaine-homocysteine S-methyltransferase [15]
	*DNMT1*	DNA (cytosine-5)-methyltransferase 1 [15]
	*DNMT3B*	DNA (cytosine-5)-methyltransferase 3B [15]
	*N6AMT1*	N-6 Adenine-specific DNA methyltransferase 1 [32]
	*PEMT*	Phosphatidylethanolamine N-methyltransferase [15]

**Table A2:** Top 15 windows of PBS in Collas.

Rank	Genes in window	Window location	PBS	Max. score position
1	*ACY3, ALDH3B2, TBX10*	11: 67400,000-67500000	1.188	23 kb Downstream *ALDH3B2*
2	*CBS, MX2, PKNOX1*	21: 44400000-44500000	1.005	Within *CBS* and *MX2*
3	*HRH1, RP11-572M11.3, SNORD112*	3: 112800000-112900000	0.993	Within *HRH1*
4	*CYP17A1, CYP17A1-AS1, WBP1L*	10: 104500000-104600000	0.965	Within *CYP17A1*,38 kb upstream of *AS3MT*
5	*TSPAN18*	11: 44700000-44800000	0.955	27 kb Upstream of TSPAN18
6	*RN7SL710P*	14:97900000-98000000	0.950	36 kb Upstream of *RN7SL710P*
7	No gene	13: 103800000-103900000	0.934	n.a.
8	*ATP2B2, ATP2B2-1T1, ATP2B2-1T2*	3: 10600000-10700000	0.929	Within *ATP2B2*
9	*PLCH1, PLCH1-AS1*	3: 155100000-155200000	0.925	Within *PLCH1*
10	*CNNM2*	10: 104700000-104800000	0.906	Within *CNNM2*
11	*CNNM2, C10orf32, AS3MT*	10: 104600000-104700000	0.906	Within *CNNM2*,15 kb downstream of *AS3MT*
12	*LHFP*	13: 40100000-40200000	0.893	Within *LHFP*
13	*CRYAA, U2AF1*	21: 44500000-44600000	0.872	Within *MX2*
14	*L1NC00856*	10: 80300000-80400000	0.871	Within *LINC00856*
15	*PDSS1*	10: 26900000-27000000	0.870	617 bp Upstream of *PDSS1*
